# Shining a Light on Colibactin Biology

**DOI:** 10.3390/toxins13050346

**Published:** 2021-05-12

**Authors:** Michael W. Dougherty, Christian Jobin

**Affiliations:** 1 Department of Medicine, University of Florida, Gainesville, FL 32610, USA; Michael.dougherty@medicine.ufl.edu; 2 Department of Infectious Diseases and Inflammation, University of Florida, Gainesville, FL 32610, USA

**Keywords:** colibactin, genotoxin, *pks*, *Escherichia coli*, colorectal cancer, DNA damage, mutation, microbiome, inflammation, *APC*

## Abstract

Colibactin is a secondary metabolite encoded by the *pks* gene island identified in several Enterobacteriaceae, including some pathogenic *Escherichia coli* (*E. coli*) commonly enriched in mucosal tissue collected from patients with inflammatory bowel disease and colorectal cancer. *E. coli* harboring this biosynthetic gene cluster cause DNA damage and tumorigenesis in cell lines and pre-clinical models, yet fundamental knowledge regarding colibactin function is lacking. To accurately assess the role of *pks*^+^ *E. coli* in cancer etiology, the biological mechanisms governing production and delivery of colibactin by these bacteria must be elucidated. In this review, we will focus on recent advances in our understanding of colibactin’s structural mode-of-action and mutagenic potential with consideration for how this activity may be regulated by physiologic conditions within the intestine.

## 1. Introduction

*Escherichia coli (E. coli*) are a group of Gram-negative facultative anaerobic bacteria isolated from multiple body sites of almost every individual, paradoxically implicated in both intestinal homeostasis and various pathologies depending on species and subspecies classification [1]. Certain *E. coli* strains, primarily from the B2-phylogroup, harbor a pathogenic island termed *pks*. This biosynthetic gene cluster is widely distributed throughout various Enterobacteriaceae and encodes for a secondary metabolite named colibactin, putatively acquired through horizontal gene transfer as part of a mobile genetic element [2]. The widespread distribution, prevalence, and evolutionary persistence of *pks* genes in this bacterial family suggest that this biosynthetic gene cluster may promote host fitness. In contrast, these compounds have deleterious effects in eukaryotic cells, inhibiting cell-cycle progression and inducing DNA damage [3,4]. Recently, the structure of colibactin has been identified [5]. Colibactin contains dual electrophilic warheads each capable of binding DNA, suggesting that colibactin causes DNA damage by inducing interstrand DNA-crosslinks [5,6,7].

The production of a genotoxic cyclomodulin by a common commensal microbe (*E. coli*) led to the hypothesis that these microbes may play a causative role in a subset of colorectal cancer (CRC) cases, as exposure to environmental genotoxins may promote specific somatic mutations implicated in a variety of cancers [8]. In murine models of colitis-associated cancer, administration of *pks^+^ E. coli* promotes tumor formation [4]. Furthermore, *pks^+^ E. coli* are identified in tumor biopsies from CRC (66.7%) and IBD cases (40%) at a higher rate than healthy individuals (20.8%) [4]. Subsequent studies utilizing in vitro models ascertained a unique mutational signature occurring after exposure to *pks^+^ E. coli* consistent with the hypothesized nucleotide binding activity of the recently proposed colibactin structure, and identified these mutational signatures in approximately 5% of colorectal cancer patients [9]. Collectively, these studies have generated a high amount of research aimed at elucidating colibactin’s mutagenic potential in the context of CRC etiology. However, colibactin itself is a highly unstable molecule and cancer development is dependent on specific biological context, leading to some debate regarding its role as an initiator of tumorigenesis [10]. Thus, a number of recent studies have focused on identifying how colibactin regulation and *pks*^+^ *E. coli* biology may influence the oncogenic capacity of the compound [11,12,13,14,15,16]. In this review, we will summarize how advances in our understanding of colibactin’s structural mode-of-action have begun to clarify the role *pks^+^ E. coli* may play in CRC, and how new findings describing *pks* regulation shed a light on how physiologic contexts may influence its carcinogenic potential. 

## 2. Colibactin Genotoxicity

Bacterial genotoxins are secondary metabolites which directly damage host DNA. Such compounds have the potential to trigger genomic instability, as cells with deficiencies in DNA repair pathways or are utilizing error-prone repair pathways have a high risk of mutational acquisition following exposure to genotoxic stressors in their environment [17]. Because genetic mutations are a hallmark of almost all cancers [18] and *pks^+^ E. coli* promote tumor formation in pre-clinical models [4], it is reasonable to theorize that colonization by microbes producing colibactin may be linked to higher cancer risk. However, until recently colibactin’s mode-of-action remained unclear. In this section, we will review how recent advances in structural characterization of this compound have helped elucidate the mutagenic potential of *pks^+^ E. coli,* providing a putative mechanism for carcinogenic phenotypes observed nearly a decade earlier. 

### 2.1. Structure and Alkylating Activity

**Figure 1:**
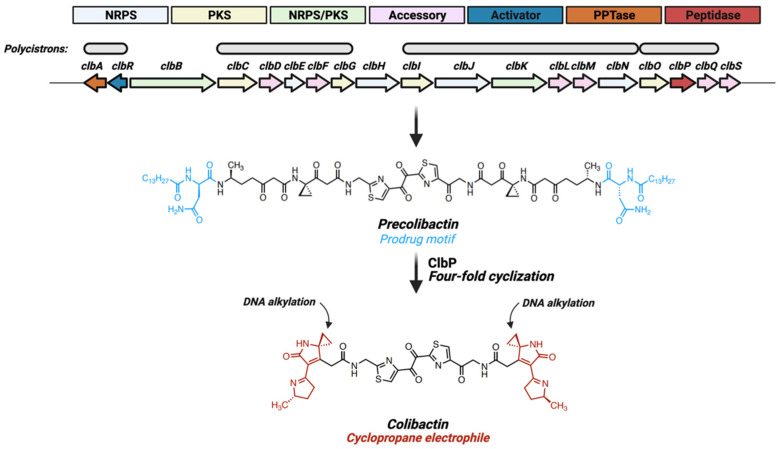
Colibactin is a product of the 54-kb *pks* gene island. The *pks* gene island consists of 19 *clb* genes transcribed in four polycistronic and three cistronic elements, encoding various non-ribosomal peptide synthetase (NRPS), polyketide synthetase (PKS), hybrid NRPS-PKS or accessory proteins. Colibactin production is regulated by the LuxR-type transcriptional activator ClbR and the pantetheinyltransferase (PPTase) ClbA. Following transcriptional activation, a biosynthetic scaffold coordinates production of a linear intermediate (precolibactin) harboring a *N*-myristoyl-D-Asn motif. The transmembrane peptidase ClbP removes these prodrug motifs, inducing spontaneous dual two-fold cyclizing events resulting in production of the bioactive colibactin molecule, characterized by two electrophilic cyclopropane warheads with high binding-affinity for adenine residues within AAWWTT nucleotide motifs.

Colibactin is a secondary metabolite produced by the 54-kb *pks* gene island encoding for a hybrid non-ribosomal peptide synthetase (NRPS)—polyketide synthetase (PKS) assembly line and accessory proteins (*clbA—S*; Figure 1) [3]. This NRPS-PKS protein assembly produces linear biosynthetic intermediates termed precolibactin which are characterized by a *N*-myristoyl-D-Asn prodrug motif (Figure 1). Precolibactins are translocated into the periplasm via the multi-drug and toxin extrusion transporter ClbM [19] and then converted to genotoxic colibactin through the removal of prodrug motifs via the membrane-bound peptidase ClbP [20]. Removal of prodrug motifs causes spontaneous four-fold cyclization of linear precolibactins to yield the bioactive colibactin structure (Figure 1) [21]. Colibactin is composed of two nearly symmetrical subunits, each containing an electrophilic cyclopropane warhead which binds adenine residues on DNA to generate interstrand cross-links (Figure 1) [5,21,22]. The incorporation of prodrug motifs in colibactin biosynthesis may be part of a self-resistance mechanism common to toxin biosynthesis pathways in multiple bacterial species [20]. However, studies utilizing *clbP* mutants to characterize various shunt precolibactins demonstrated that a subset of these molecules, in the presence of copper, generate interstrand cross-links (ICLs) in linearized plasmid DNA [7], a phenomena still not fully understood. For several years, colibactin’s structure and mode-of-action remained elusive due to its resilience to traditional isolation techniques and high instability. Recent studies have utilized DNA-adducts formed during exposure of *pks*^+^ bacteria to linearized plasmids [5] or within gnotobiotic mice [22] to characterize the structure of DNA-bound colibactin, providing the first insight into colibactin’s molecular structure and identifying DNA-reactive electrophilic sites capable of alkylating DNA by ring-opening addition [5]. *In silico* modeling utilizing structures derived from adductomics suggest colibactin has a high binding affinity for adenine rich motifs (AAAATT) within the DNA minor groove [23]. These findings support colibactin’s proposed activity as a DNA cross-linking agent and have helped clarify the molecular mechanisms of colibactin-associated genotoxicity. The next section will address mammalian host response mechanisms associated with *pks*^+^ *E. coli* exposure.

### 2.2. Genotoxic Activity in Mammalian Cells

**Figure 2:**
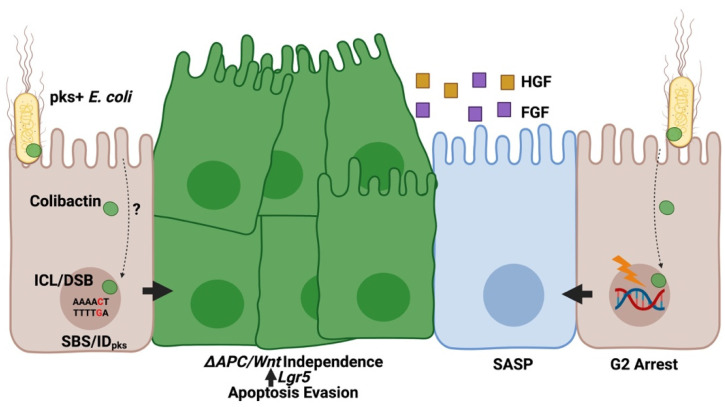
*Pks*^+^ *E. coli* promote tumor formation in the colonic epithelium. During infection with *pks*^+^ *E. coli*, colibactin molecules translocate to the host nucleus via an undetermined mechanism, where the compound generates inter-strand crosslinks (ICLs) in adenine-rich nucleotide motifs and double-strand DNA breaks (DSBs). Errors during DNA repair following *pks*-induced damage result in the accumulation of a specific mutational signature characterized by T > N single base substitutions (SBS_pks_) or insertion/deletions of varying length (ID_pks_). Exposure to *pks*^+^ *E. coli* induces oncogenic phenotypes characterized by enhanced proliferation and *Wnt* independence. Unrepaired lesions cause cell-cycle arrest. Arrested cells adopt a senescence-associated secretory phenotype (SASP) resulting in enhanced growth factor production (hepatocyte growth factor [HGF], fibroblast growth factor [FGF]) which promote proliferation of nearby cells.

Several studies have demonstrated the genotoxic property of *pks*^+^ *E.* *coli* and have identified signaling pathways associated with this cellular response. For example, *E. coli* harboring a bacterial artificial chromosome encoding the complete *pks* gene island (BAC_pks_) activate classical ataxia telangiectasia (ATM) and Rad53 related (ATR) dependent DNA damage responses (DDR) in infected HeLa cells, characterized by phosphorylation of checkpoint kinase 2 (CHK2), replication protein A (RPA), γH2AX, and the accumulation of phosphorylated cyclin dependent kinase 1 (CDC2) resulting in G2-cell cycle arrest (Figure 2) [3]. *Pks*-induced DDR activation in HeLa cells promotes recruitment of the interstrand cross-link (ICL) repair protein FANCD2 to phosphorylated γH2AX sites [6], suggesting involvement of the Fanconi-anemia (FA) pathway in repairing colibactin-generated ICLs. While these results are consistent with colibactin’s proposed alkylating activity, several other studies suggest infection with *pks*^+^ *E. coli* also induces double-strand breaks (DSBs), which can be directly visualized by neutral comet assay after infection [3]. In CHO cells, exposure to *pks*^+^ *E. coli* causes increased recruitment of the non-homologous end joining repair protein Ku80 [24], a pathway primarily involved in the resolution of DSBs rather than ICLs. Structural elucidation of colibactin implies that the compound is likely not involved in the direct formation of DSBs, suggesting that DNA breaks may instead be the product of endogenous changes occurring after the formation of alkylated lesions generated by colibactin-mediated cross-linking. Activation of FA pathways may indirectly cause DSBs, as cells excise cross-linked DNA using homologous cellular pathways (e.g., γH2AX) before undergoing repair [25]. Alternatively, the internalization of certain *pks*^+^ adherent invasive *E. coli* (AIEC) strains may promote the production of reactive oxygen species (ROS) which cause DSBs [26] expected to trigger the apurinic/apyrimidinic endonuclease system (APE1). It is unclear if ROS production is dependent on *pks* or simply a response to bacterial internalization which may exacerbate DNA damage caused by colibactin.

Perhaps the most cryptic aspect of colibactin’s genotoxic mechanisms is the method by which these molecules translocate from the bacterial periplasm to the host nucleus (Figure 2). It is known that cell-to-cell contact is necessary for genotoxicity after *pks*^+^ infection in vitro, as separation of mammalian cells and bacteria by cell-impermeable membranes attenuates colibactin’s cytotoxic effects [3]. It seems likely that the compounds instability may prevent remote delivery by pathogenic bacteria but remains unclear if specific secretion systems are necessary to deliver colibactin to host cells. So far, no essential transporters for *pks*^+^ genotoxicity have been identified. Interestingly, early experiments inhibiting bacterial internalization did not attenuate BAC_pks_ genotoxicity [3]. In contrast, a clinical *pks*^+^ AIEC isolate from a CRC patient was shown to invade and replicate within epithelial cells, with a concomitant increase in tumor formation [27]. In murine models administered this strain, oncogenic activity could be attenuated by either effective host autophagy or mutation of the *pks* editing thioesterase *clbQ* required for colibactin production [27], suggesting a potential association between intracellular invasion and *pks*^+^ AIEC’s tumorigenic efficacy. However, it remains unclear if this internalization enhances colibactin’s effects or is simply a byproduct of lowered host defense (in this case, genetically modified autophagy-deficient mice) while autophagy promotes DDR through a different mechanism. Since release of bacterial outer member vesicles have been linked to DNA damage [28], it would be interesting to investigate this mode of bacterial-host communication in *pks*^+^ *E. coli* mediated genotoxicity.

### 2.3. Pks-Induced Mutagenesis

In mammalian cells, exposure to *pks*^+^ *E. coli* leads to the accumulation of chromosomal aberrations and aneuploidy, suggesting colibactin exposure may be linked to oncogenic mutation [24]. Acute exposure of human colonic organoids to *pks*^+^ *E. coli* induces functional mutations related to the p53 and Wnt signaling pathways [29]. Importantly, colonic organoid cultures continue to proliferate in the absence of the traditionally essential growth factors Wnt3a and CHIR99021, both regulators of the adenomatous polyposis coli (*APC*) signaling pathways mutated in 80% of CRC cases [29]. These derived Wnt-independent cultures expressed higher stem-cell transcriptional signatures (e.g., Lgr5, Fzd7, Sox9) and avoided p53-mediated apoptosis following administration of the p53 pathway activator Nutlin-3a, providing direct evidence of oncogenic mutation following *pks*^+^ *E. coli* infection (Figure 2) [29]. Two independent groups identified mutational signatures arising from *pks*^+^ *E. coli* infection in vitro [9,23]. Pleguezuelos-Manazno, Puschhof, and Huber et al. [9] performed whole-genome sequencing of clonal organoids after chronic exposure to *pks*^+^ *E. coli*. These investigators identified a mutational signature characterized by a high number of single-base substitutions (SBS-*pks*, T > N) and less common short insertion-deletions (ID-*pks*) within AT-rich DNA regions. Similarly, Dziubanska-Kusibab et al. [23] demonstrated specific enrichment of SBSs within AT-rich pentanucleotide sequences located in the DNA minor groove following short-term exposure of Caco-2 cells to *pks*^+^ *E. coli*. During chronic infection, a majority of SBS-*pks* motifs occurred within the coding DNA strand and mutations matching either SBS-*pks* and ID-*pks* motifs were identified in 5% and 4.4%, respectively, of CRC tumors from an independent database of 2208 predominately primary sites [9]. In these cases the gene with the highest number of mutations matching the *pks*-target motif was the *APC* gene (5.3%), which is frequently mutated in CRC cases [9]. Interestingly, the incidence of SBS/ID-*pks* motifs in CRC cases is much lower than the incidence of *pks*^+^ *E. coli* identified from colorectal tumors (67% [4]), further implying that the carcinogenic capacity of these microbes is derived from the combinatory production of colibactin and other aspects of intestinal ecology (see commentary [30]). Collectively, these findings provide direct evidence of the mutagenic potential of colibactin in the context of CRC (Figure 2). Importantly, presence of *pks*^+^ bacteria alone is likely insufficient for cancer development, and the colibactin mutational signature is found in a large number of healthy individuals as well as CRC patients [31], suggesting typically commensal *pks*^+^ bacteria may only exert a carcinogenic influence under specific conditions.

## 3. Colibactin Activity in Physiologic Context

In order to exert a mutagenic influence on the host epithelium in vivo, experimental evidence suggests *pks^+^ E. coli* likely requires two physiologic factors, namely: (1) transcriptional activation of all *clb* genes (besides *clbS*), and (2) cell-to-cell contact, i.e., a close association between colonizing *pks^+^* strains and host epithelial cells [3]. Bacterial metabolism and biosynthesis of specific compounds is regulated through transcriptional mechanisms influenced by metabolic conditions in the environment [32,33,34]. Thus, an understanding of how *pks^+^ E. coli* regulate colibactin with respect to environmental condition, particularly during opportunistic expansion near the host epithelium, is critical to assessing the carcinogenic potential of these bacteria. In this section, we will summarize the current framework of colibactin regulation and how its genotoxic potential may be functionally influenced by environmental condition. Finally, we will summarize a newly emerging research area focused on characterizing colibactin’s effects extending beyond direct DNA damage.

### 3.1. Metabolic Regulation of pks Genes

**Figure 3:**
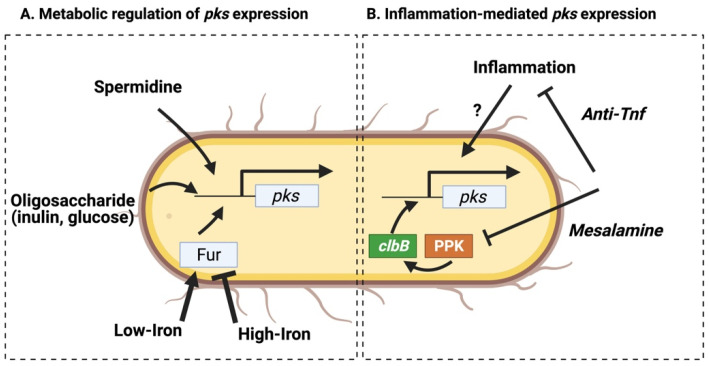
Transcriptional regulation of *clb* genes. (**A**) Transcription of the regulatory *clb* genes (*clbA* and *clbR*) is increased in low-iron or oligosaccharide rich environments. The endo- or exogenously derived polyamine spermidine is necessary for *pks* transcription. (**B**) Inflammation promotes the transcription of several individual *clb* components. Administration of anti-inflammatory drugs such as anti-TNF attenuate these effects and limit *pks*-associated tumorigenicity *in vivo*. Anti-polyphosphate kinase (PPK) inhibitors downregulate *clbB* expression and the genotoxicity of *pks*^+^ *E. coli*.

The *pks* operon consists of four polycistronic transcripts (of seven total) oriented in a single direction, with the exception being a single polycistron encoding the pantetheinyltransferase (PPTase) (*clbA*) and the LuxR-type transcriptional activator (*clbR*) (Figure 1) [35] that promote the expression of downstream *pks* genes [11]. Transcription of these regulatory elements may govern colibactin production, and their expression levels are directly influenced by environmental metabolites (Figure 3a). With respect to the dominant intestinal taxa (e.g., Bacteroidetes, Firmicutes), *E. coli* have relatively limited glycolytic capacity, instead relying on an abundant siderophoric repertoire to opportunistically thrive in iron-limited conditions [36]. These pathways are controlled by the ferric iron uptake regulator (Fur) that is activated in low-iron conditions and binds directly to the promoter region of the *pks* gene *clbA* [15], which in turn acts as both an additional siderophore and an initiator of colibactin biosynthesis [16]. The regulatory *pks* gene *clbR*, which acts as a transcription factor for *clbB*, is similarly upregulated in low iron conditions [11]. As expected given these findings, when *pks*^+^ *E. coli* are cultured in high-iron conditions production of colibactin is inhibited [12]. Additionally, the colibactin transmembrane peptidase ClbP is required for production of the siderophore microcin in *pks*^+^ *E. coli* Nissle 1917 (EcN), and is necessary for colonization in the presence of other opportunistic pathogens such as *Salmonella* Typhimurium [13]. Thus, *pks* genes may provide an evolutionary advantage to *E. coli* strains by enhancing iron-scavenging capabilities by acting as or contributing to the maturation of critical siderophores. 

Other metabolites derived endogenously from *pks*^+^ *E. coli* or the host diet similarly alter transcription of *clb* genes. Spermidine, a polyamine produced during bacterial metabolism or scavenged from the environment is necessary for the production of colibactin [14]. Administration of oligosaccharide prebiotics, such as inulin or glucose, similarly enhance *clbA* transcription and *pks*^+^ genotoxicity [37]. In contrast, metabolites promoting *pks* transcription can be attenuated by inhibitory factors (such as ferrous sulfate [37]), suggesting that regulation of colibactin production is thus intrinsically linked to metabolic conditions within the intestinal lumen or the mucosal lining. These findings are particularly relevant when considering how the carcinogenic activity of *pks*^+^ *E. coli* may be enhanced in colitis or CRC patients, who often present with anemia [38] or other disruptions to metabolic homeostasis which may alter *pks* transcription. However, since direct measurement of colibactin level is currently unavailable, the consequences of transcriptional changes in individual *pks* genes on cellular genotoxicity in vivo is unclear.

### 3.2. Inflammation

Inflammatory bowel disease (IBD) creates a pro-neoplastic environment within the colonic epithelium [39] that promotes dysplasia and the development of colitis-associated cancer (CAC) [40]. Inflammation heavily modulates microbial balance in the gut, typically associated with increased Proteobacteria/Enterobacteriaceae/*E. coli* prevalence. For example, chemically-induced inflammation following dextran sodium sulfate (DSS) treatment reduces populations of anaerobic Bacteroidetes by approximately 70% while increasing levels of various aerobic species by approximately 25% [41]. In this acute model, the abundance of a nonpathogenic *E. coli* strain doubled in DSS treated mice, and colonization in *E. coli*-naïve mice corresponded with a decrease in mucosal Bacteroidales colonization [41]. Several epidemiological studies have established a link between IBD or CRC and increased mucosal colonization by *pks*^+^ *E. coli,* accounting for as much as 13% (IBD) or 26% (CRC) of all *E. coli* strains isolated from these patients [4,42,43]. These findings suggest tumorigenesis may be driven, at least partially, by selective enrichment of *pks*^+^ strains within the colonic mucosa. This hypothesis is borne out by studies in murine models of CAC utilizing azoxymethane treated *Il10^−/−^* mice, showing that the presence of *pks*^+^ *E. coli* promotes DNA damage and neoplastic transformation under inflammatory conditions [4] abrogated in Rag2^−/−^ mice with no inflammatory response [44]. In similar models of CAC, limiting *pks*^+^ *E. coli* colonization during inflammation by inhibiting nitrate reductase activity abrogates *pks*-associated tumorigenesis [45]. However, the current clinical impracticality of eradicating *E. coli* populations in colitis patients renders this an unlikely preventative treatment option to limit tumor progression in IBD patient.

Alternatively, elevated *pks* expression in inflamed tissue may be attenuated as a byproduct of IBD treatments which dampen inflammatory cascades or directly inhibit *clb* gene transcription. In murine CAC models gavaged with *pks*^+^ *E. coli*, inflammation promotes transcription of multiple *clb* genes involved in colibactin biosynthesis during tumor initiation [44]. A recent study by Yang et al. [46] demonstrates that anti-TNF treatment prevents colonic inflammation and subsequent tumor development in both chemically-induced (DSS/*Apc^Min/+^*) and spontaneous (*Il10^−/−^ Apc^Min/+^*) CAC models by modulating microbiota composition and transcription, while co-housing anti-TNF treated with control mice prevented these microbial change and inhibited the anti-tumor effects of TNF neutralization [38]. Interestingly, targeting inflammation with anti-TNF antibody did not alter *pks*^+^ *E. coli* colonization level, pointing to a change in microbial activity [46]. Related to this, the anti-inflammatory drug mesalamine inhibits the microbial enzyme polyphosphate kinase (PPK), sensitizing bacteria to oxidative stress and inhibiting proliferation *in vitro* [47]. In a small cohort of human test subjects, mesalamine reduced microbial polyP accumulation, suggesting PPK inhibition reduces microbial metabolism in a clinical setting [47]. One would expect that these effects would extend to virulence associated with *pks^+^ E. coli*, and the evidence suggests mesalamine directly inhibits colibactin production and ICL formation in vitro [48]. These studies suggests that anti-inflammatory treatment may limit the risk of CRC by acting on the host to limit inflammation-induced dysplasia while simultaneously limiting production of carcinogenic toxins released by opportunistically pathogenic *pks*^+^ *E. coli* (summarized in Figure 3b). Thus, a more nuanced understanding of how modulating the inflammatory environment alters colibactin concentrations may help direct treatment options in IBD patients with high levels of colibactin-producing bacteria, who may be at a higher risk for cancer progression.

### 3.3. Mucosal Adherence and Biofilm Formation

**Figure 4:**
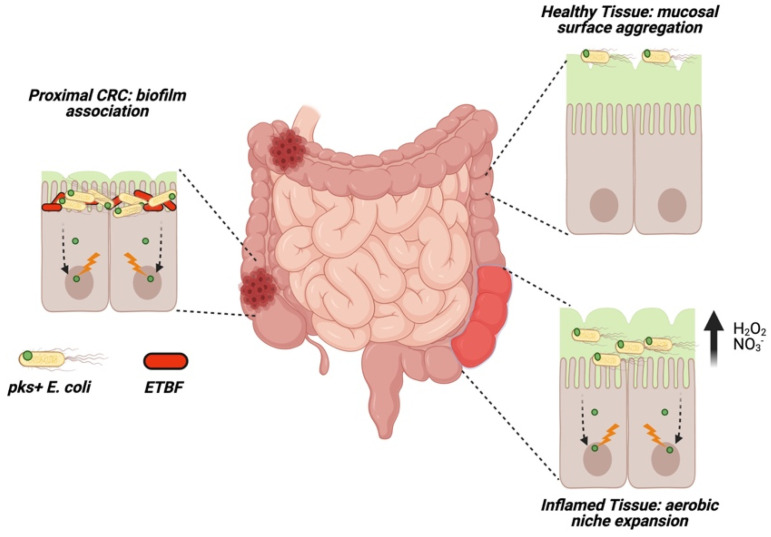
The oncogenic capacity of *pks*^+^ *Escherichia coli* is enhanced by increased mucosal invasion and biofilm association. In healthy tissue, *E. coli* typically aggregate at the mucosal surface. In biofilm-covered CRCs, *pks*^+^ *E. coli* form biofilms in cooperation with enterotoxigenic *Bacteroides fragilis* (ETBF), increasing depth of mucosal invasion. During inflammation, a microaerobic niche formed within the mucosa (derived from host nitric oxides or peroxides) facilitates the expansion of mucosal *E. coli* populations.

In vitro evidence that cell-to-cell contact is necessary for colibactin’s genotoxic activity suggests that delivery of the molecule in vivo may necessitate *pks*^+^ bacteria somehow bypassing the protective mucosal barrier to interact directly with the host epithelium. *E. coli* possess various fimbral adhesins which promote binding and internalization at the colonic mucosa [49], suggesting *pks*^+^ *E. coli* may colonize and invade the mucosa increasing potential direct contact with epithelial cells. In tumor tissue, this is often the case: biopsies from CRC patients show a higher level of mucosally-invasive *E. coli* relative to normal adjacent tissue where *E. coli* aggregate at the mucosal surface (Figure 4) [50,51], and tumors with high microsatellite instability exhibit higher rates of mucosally-invasive *E. coli* [26]. Clinical *E. coli* isolates exhibiting high mucosal invasion show high binding affinity to mucus secreting cells in vitro, with *pks*^+^ strains inducing high levels of DNA damage exacerbated by mucosal disruption [52]. During inflammation, host production of nitric oxides and reactive oxygen species increases mucosal oxygenation, creating a microaerobic niche in which *E. coli* preferentially colonize (Figure 4) [53,54]. These changes may promote proliferation of mucosal *pks*^+^ *E. coli* species during inflammation, providing a mechanism for colibactin induced oncogenesis in CAC.

At the mucosal interface, aggregating bacteria can encapsulate themselves in a self-secreted extracellular matrix (i.e., biofilm) to avoid perturbations from host defenses or the intestinal environment [55]. Biofilm structures are often found in patients with intestinal disease, and promote intramucosal invasion [56]. In *E. coli* strains producing genotoxins, biofilm association increases mucosal invasion depth which may facilitate the delivery of virulent small molecules to host epithelial cells (Figure 4). In two geographically distinct cohorts from the USA and Malaysia, right-sided (proximal) CRC cases almost universally (89%) harbored biofilms both at the tumor site and throughout the normal mucosa [57]. While bacterial diversity in normal and tumor tissue did not differ, biofilm-covered CRCs exhibited higher bacterial invasion into the underlying tumor tissue [57].

Several studies have used a combination of *in-situ* hybridization and 16S rRNA gene sequencing to investigate the composition of mucosal biofilms in CRC patients, frequently identifying Proteobacteria in mucosal biofilms from both tumor and normal adjacent tissue, with a high prevalence of *pks*^+^ *E. coli* (68%) in proximal CRC biofilms [58,59]. Biofilms harvested from both normal and tumor tissue from CRC increase mucosal invasion by gut microbes and induce a higher tumor burden in murine models [60], suggesting some element of core biofilm structure, a commonly distributed biofilm-associated species, or a combination of these factors, promotes carcinogenesis. A causative role for *pks*^+^ *E. coli* seems likely given their ubiquitous distribution throughout biofilms isolated from CRC patients. Co-infection with the biofilm initiating strain enterotoxigenic *Bacteroides fragilis* (ETBF) and *pks*^+^ *E. coli* in specific pathogen free (SPF) mice demonstrate that biofilm association promotes mucosal invasion, expansion of *pks*^+^ *E. coli* populations, and a concomitant increase in γH2AX histological staining and tumor burden relative to monoassociation [58]. ETBF may further promote interaction between *pks*^+^ *E. coli* and host epithelial cells by degrading the protective mucus layer through an undetermined mechanism (see commentary [61]), minimizing a crucial barrier for *E. coli* adherence. Importantly, deletion of *B. fragilis* toxin (ETBF) or colibactin (*E. coli*) diminished tumor burdens, suggesting the existence of a cooperative network between these toxins [58]. However, the use of SPF mice in these experiments makes it difficult to distinguish if additional bacteria are involved in biofilm initiation and tumor development.

Altogether, these findings suggest that *pks*^+^ *E. coli* association with microbial biofilms enhances colibactin’s carcinogenic activity, possibly by increasing proximity to host epithelial cells and enhancing total abundance of these bacteria within the mucosa. In these scenarios, normally innocuous genotoxins secreted by the bacteria may occur in elevated doses near replicating host cells resulting in carcinogenic activity and CRC initiation or progression.

### 3.4. Modulation of Tumor Microenvironment

While colibactin can directly promote oncogenic transformation by DNA alkylation and subsequent mutagenesis in epithelial cells [6,9,23,29], its role in CRC progression extends beyond these activities by helping to establish a pro-carcinogenic environment supporting tumor growth [62,63,64]. After *pks*-induced DNA damage, cells may undergo apoptosis, transformation, or senescence. Adoption of a senescent state induces a senescence-associated secretory phenotype (SASP) characterized by increased secretion of various growth factors that promote proliferation of nearby cells (Figure 2) [62,63]. These changes may occur near cells that have acquired *pks*-generated mutations in the *APC* or *p53* pathways after colibactin exposure [29], further promoting unrestricted growth and tumor proliferation. A recent study demonstrated that the *pks*^+^ *E. coli* strain 11G5 translocates to mesenteric lymph nodes in *Apc*^Min/+^ mice, with a concomitant reduction of some cytotoxic T cell lineages and an increase in regulatory T cells within infected lymph nodes [64]. Furthermore, levels of cytotoxic T cells are reduced within the colonic mucosa of 11G5-colonized mice and invasive margins from tumor biopsies from CRC patients colonized by *pks*^+^ *E. coli* [64], suggesting that colibactin-producing microbes may create a pro-carcinogenic environment within the gut by modulating immune cell activity. Collectively, these observations suggest that colibactin-producing *E. coli* may create a “perfect storm” of tumorigenic potential, simultaneously promoting oncogenic transformation in epithelial cells and generating populations of bystander tumor-promoting cells while restricting immune activation. 

Colibactin may also influence interactions between *pks*^+^ *E. coli* and other intestinal bacteria, altering the complex ecological networks observed within gut microbiota communities [65]. Shunt precolibactins have been shown to have mild inhibitory effects on the growth of a common probiotic, *Bacillus subtilis*, in vitro [66]. The effects of colibactin on microbial communities in vivo remain poorly characterized, but at least one study has directly demonstrated that colonization by *pks*^+^ *E. coli* in murine models reduces the relative abundance of Firmicutes and Clostridia [67], taxa which represent a significant proportion of microbes in healthy individuals [68]. Disruption of the ratio of Firmicutes to Bacteroidetes has been used as a marker of various metabolic disorders [69] and a general measure of dysbiosis. The exact nature of how colibactin production may alter transcriptional regulation or abundance of gut microbes is not well understood. However, the relevance of microbial dysbiosis in the etiology of various cancers is well-defined [70,71,72]. Future studies focusing on how colibactin influences multi-kingdom interactions in the gut may elucidate novel pathways by which *pks*^+^ bacterial colonization deleteriously affects their host.

## 4. Conclusions

Overrepresentation of *pks*^+^ *E. coli* in CRC cases has led to the hypothesis that these bacteria may directly promote tumorigenesis. Several biological characteristics of colibactin supports a role for this secondary molecule in carcinogenesis. First, colibactin contains two electrophilic cyclopropane warheads that bind adenine-rich motifs in host DNA, creating inter-strand cross-links that stall cell cycle progression and activate DNA damage response pathways. Infection with *pks*^+^ *E. coli* causes somatic mutation that induces oncogenic transformation in colonoids and promotes tumor development in vivo. However, the prevalence of *pks*^+^ *E. coli* colonization far exceeds the observed proportion of individuals carrying mutational signatures attributed to the genotoxin, suggesting physiological context dictates the compounds genotoxic activity. This raises an important question: is the presence of *pks* genes enough to produce colibactin with functional genotoxic effect on host epithelium? An interesting case-study can be made by investigating the genotoxic potential of the commercially available probiotic strain *E. coli* Nissle 1917 (EcN), that has a long history of use as a beneficial probiotic [73] despite carrying the *pks* biosynthetic gene cluster [3]. Counterintuitively, the *pks* peptidase ClbP is actually essential for the antibacterial activity of EcN, contributing to the maturation of siderophores that enable this probiotic to outcompete pathogenic bacteria [13]. Whether or not EcN infection results in DNA damage is debated. While one recent study reported no genotoxic effects of EcN were observable in cell lines or murine models [74], others have demonstrated genotoxicity in mammalian cell lines attenuated by *clbA* mutation [13]. Thus, more work is necessary to determine if these strains produce physiologically active colibactin, and if long-term intake of this probiotic may increase cancer risk.

**Figure 5:**
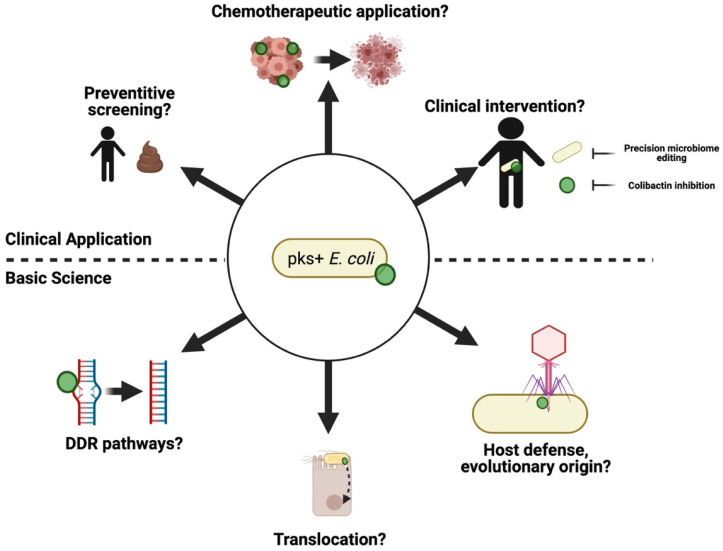
Future areas of research involving *pks*^+^ *E. coli*.

*Pks*^+^ bacteria may have relevance to cancer outside of the intestine. These bacteria frequently colonize extraintestinal sites and colibactin mutational signatures have been reported in a subset of patients with head and neck, urinary tract, neuroendocrine tumors, and ovarian cancer [9]. A higher concentration of colibactin biosynthetic byproducts in the urine of patients with urinary tract infection (UTI) relative to healthy individuals, and an archetypal UTI strain of *pks^+^ E. coli* causes DNA damage within the regenerative compartment of the bladder after transurethral infection in mice, suggesting a potential role for these microbes in bladder cancer [75]. Two separate studies currently published as pre-prints have identified a high proportion of *pks*^+^ *E. coli* in association with DNA damage in biopsies from prostate cancer patients [76] and *pks*^+^ *Klebsiella pneumoniae* in a subset of hospital patients with liver abscess [77]. These findings suggest that colibactin may play a role in a variety of cancers beyond CRC. Although solid evidence supports the case for colibactin as a carcinogen, the oncogenic potential of *pks*^+^ *E. coli* may be influenced by many factors altering colibactin production and localization within the intestinal tract. Colibactin’s instability suggests it may only exhibit oncogenic activity while in close association with host epithelial cells, and limits mechanistic studies aimed at studying the effects of the toxin without influence from other aspects of bacterial infection or unrelated virulence factors. Because all colibactin-related studies utilize *pks* or *clb* mutants (e.g., [3,5,6,13]) which may influence other biosynthetic pathways in ways we do not completely understand [78], it is difficult to attribute phenotypes directly to colibactin. Recent structural characterization of colibactin may facilitate the production of synthetic compounds which can be used to answer questions specifically regarding structure-activity relationship. These synthetic compounds would allow for more comprehensive and accurate screening methods capable of identifying pathways involved in DNA repair following exposure to *pks*^+^ *E. coli*, or for the derivation of targeted therapeutics which inhibit genotoxic activity by directly inhibiting colibactin’s mechanism-of-action. Furthermore, it remains unclear how this large transitory molecule migrates from the bacterial periplasm to the host nucleus or which DNA repair pathways eukaryotic cells use to repair cross-links formed after colibactin exposure. Future studies should focus on clarifying fundamental aspects of colibactin’s biology such as this, which remain mysterious and may inform future therapeutic approaches aimed at reducing *pks*-associated cancer risk. Pertinent research questions and potential clinical applications involving *pks*^+^ *E. coli* are summarized in Figure 5.

The most evident question is related to colibactin’s function within the microbiota. What biological advantage is gained by *E. coli* strains carrying the *pks* island? One would assume that the biosynthetic gene cluster confers a fitness advantage, but data supporting this function are limited. The extent of colibactin’s activity on other microbes (bacteria, fungi, archaea, and viruses) inhabiting the intestine is unclear at best. It is intriguing to note that certain secondary metabolites generated from biosynthetic gene clusters are implicated in bacteria anti-phage defense response [79], thereby favoring fitness to bacteria carrying this weaponry. Whether the secondary metabolite colibactin enhances bacterial fitness through anti-phage responses is currently unknown. On the flip side, colibactin analogs may find applications as chemotherapeutic agents. For example, the NPRS/PKS molecule bleomycin, which induces DNA damage is currently used as anti-cancer drug for various form of cancers [80]. There is a long road ahead before colibactin finds application in chemotherapy and for now, all efforts are focused on its cancer promoting ability.

Evidence suggests that *pks*-derived mutational signatures are acquired during childhood [9,31] and thus may contribute towards oncogenic transformation which does not appear for decades, until additional factors promote tumorigenesis. The ubiquity of B2 phylogroup *E. coli* in the human microbiome, especially at early age [81], raise the concern of long-term consequences on DNA integrity in asymptomatic hosts. However, until further clinical evidence assigns a clear carcinogenic label to *pks*^+^ *E. coli*, the need for childhood screening is nonexistent. If this moment ever arises, the challenge would be to design precision microbiome interventions since antibiotic approach would likely do more harm than good in this young population. Engineered probiotics [82] may be designed to specifically target *pks*^+^ microbes or produce metabolites which directly interrupt colibactin activity. Such small molecule inhibitors may come from a self-resistance component of the *pks* island itself, the cyclopropane hydrolase ClbS, which disrupts active cyclopropane sites in colibactin [83,84]. Phage screening may identify viruses capable of targeted depletion of specific microbial species [85], and screening *pks*^+^ isolates from individuals against phage libraries may allow for the creation of personalized phage cocktails with high activity for patient-specific strains. Such an approach has been used in pre-clinical model of CRC driven by *pks*^+^ *E. coli* [86]. In addition, predatory bacteria against AIEC such as *Bdellovibrio bacteriovorus* could represent another means to selectively deplete *pks*^+^ *E.coli* from a complex community [87]. In conclusion, it has been a remarkable journey from *pks*/colibactin’s discovery to molecular structure characterization and potential in vivo function. Yet, much remains to be addressed regarding this fascinating molecule and the years to come promise to be exciting for this field of research. 

## Data Availability

No new data were created or analyzed in this study. Data sharing is not applicable to this article.
